# Devising and Evaluating an Adaptive Static-Automated Perimetry Test for Children: A Feasibility Study

**DOI:** 10.1007/s44402-026-00049-9

**Published:** 2026-03-16

**Authors:** Andrew Turpin, Vanessa TS Tang, Siavash Salmanzadeh, Elisse Higginbotham, Allison M. McKendrick

**Affiliations:** 1 Lions Eye Institute, Perth, Australia; 2 School of Population Health, Curtin University, Perth, Australia; 3 Department of Optometry & Vision Science, School of Health and Clinical Sciences, University of Western Australia, Perth, Australia

**Keywords:** Paediatric ophthalmology, Perimetry algorithm, Visual function

## Abstract

**Purpose:**

To assess the feasibility of a Static Automated Perimetry in children test (cSAP) designed to return clinically useful information about a visual field when stopped after any number of presentations while testing the visual fields of children.

**Method:**

The cSAP test was engineered with specific location selection, test logic and data presentation as the core of the perimetric procedure. Some strategies were added to engage children in the test such as fixation markers that changed shape and colour, splitting the test into ‘levels’ and a small visual and audio reward at the end of each level. The method was run dichoptically on the Topcon Tempo Perimeter using the Open Perimetry Interface. This is a report of a validation study on 10 adults with visual field loss, looking at the differences between the final estimated field and the fields reported after each presentation, as well as comparing to a simulated baseline procedure with the same test logic but random location selection. Additionally, the experience of using the method to test 11 children aged 4–9 years is also reported, with six being retested 3 months later.

**Results:**

Stopping cSAP after any number of presentations always gave a better estimate of the final field than the baseline method in the adult eyes, although four of the 10 differences were small. The youngest child, 4 years of age, had difficulty focussing on the test to get a useful field, but generally all other children engaged in the task and returned a sensible visual field result. There was an obvious learning effect across the re-tested younger children.

**Conclusion:**

cSAP is a feasible method for testing children or others who may not complete a standard visual field test, with design advantages over conventional SAP tests for this purpose.

Key Points

- A new, engaging perimetry test has been developed that can be stopped at any time and still returns clinically meaningful measures of the visual field.
- The perimetry test was evaluated in adults with known visual field defects and shown to enable better estimates of the visual field if interrupted than standard visual field procedures.
- Children as young as 4 years of age can perform this Standard Automated Perimetry test.

## Introduction

Testing the visual field using perimetry is the clinical standard for the evaluation of vision associated with the diagnosis and monitoring of several eye diseases, in addition to neurological disorders affecting the visual pathways. One of the most common forms of perimetry is static automated perimetry (SAP), which presents small white targets on a 10 cd/m^2^ background. The test-taker responds by pressing a button when they see a stimulus. SAP requires the individual to maintain central fixation and concentration for the duration of the test, which typically takes around 5 min per eye. For able-bodied adults, this is a reasonable expectation, but for other segments of the population, SAP is too challenging to be used regularly in a clinical setting [1, 2]. In particular, young children may have trouble understanding the task, maintaining attention and fixation and find devices that are designed around adult forms uncomfortable. Previous literature suggests that children are capable of completing adult versions of SAP reliably from around age 8 years, depending on their maturity [3], but may be able to complete testing from as young as 5 years of age with non-standard training and familiarisation protocols [4]. An improved ability to measure visual fields reliably in children with conditions such as paediatric glaucoma and certain cortical disorders that may impact on visual pathway patency has the potential to improve clinical care.

This paper reports on a feasibility study of a new test designed to address some of these limitations: i.e., a SAP test that is more suitable and engaging for children. The hardware chosen was the Tempo perimeter (Topcon Healthcare Inc. (topconhealthcare.com), also marketed as the imoVifa, CREWT Medical (crewt.co.jp)). The small footprint and dichoptic testing features of the Tempo [5] were chosen to be more ergonomic for children than traditional bowl perimetry. Custom software was developed with a testing algorithm that can return useful results even when the test is terminated early, and includes features designed to increase task engagement. To validate the algorithm, it was run on 10 adults with varying degrees of visual field loss (as defined by their Humphrey Field Analyser visual field tests, Carl Zeiss Meditec, zeiss.com) and examined how the test performed relative to simulated performance using a standard test algorithm. Performance was also evaluated on 11 children between 4 and 9 years of age, as a proof-of-concept study.

## Methods

### Hardware

The prototype visual field test (with custom software) was implemented using the Tempo visual field analyser. The Tempo was chosen due to its small footprint design making it ideal for child use. Also, it allows dichoptic testing, thereby removing the need to patch one eye of the test-taker. The Tempo uses high-definition transmissive liquid crystal displays and high-intensity light-emitting diodes to enable SAP testing comparable to the Humphrey Field Analyzer, with a background luminance of 31.4 apostilbs and a maximum luminance of 10,000 apostilbs [5].

**Fig. 1:**
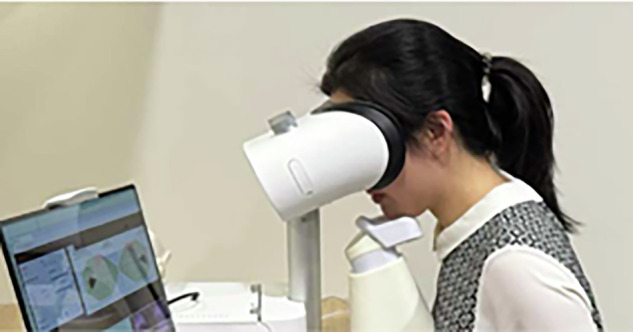
The iMoVifa (crewt.co.jp) visual field testing device with custom software display for “kids perimetry”.

Further, it is a screen-based perimeter which allows display of graphics and other elements to assist engaging children in the task. The Tempo includes an implementation of the Open Perimetry Interface (OPI) [6, 7] which allows custom programming of stimuli and responses (Fig. 1). The particular implementation of the OPI is built on top of the Java Open Visual Psychophysics toolbox [7] which allows easy display of user-defined images on the screen, and collection of responses through the button press of the device. In this study, the OPI was used to display Goldmann Size III stimuli for 200 ms, using the test logic described below.

### Software

One of the key requirements of the test for children, which we dub cSAP for convenience, was to be able to stop the test at any time and still have some clinically meaningful measure of the visual field. To this end, cSAP encompasses three main ideas: (1) careful selection of the spatial test location of the first 20 presentations; (2) a fast test that does not spend time attempting to refine threshold estimates between values of 2 to 20 dB and (3) reporting the results after each stimulus presentation in a whole visual field format to enable clinical interpretation even if the test is cut short. Each idea is described in turn in the next three sections.

### Location Selection

**Fig. 2:**
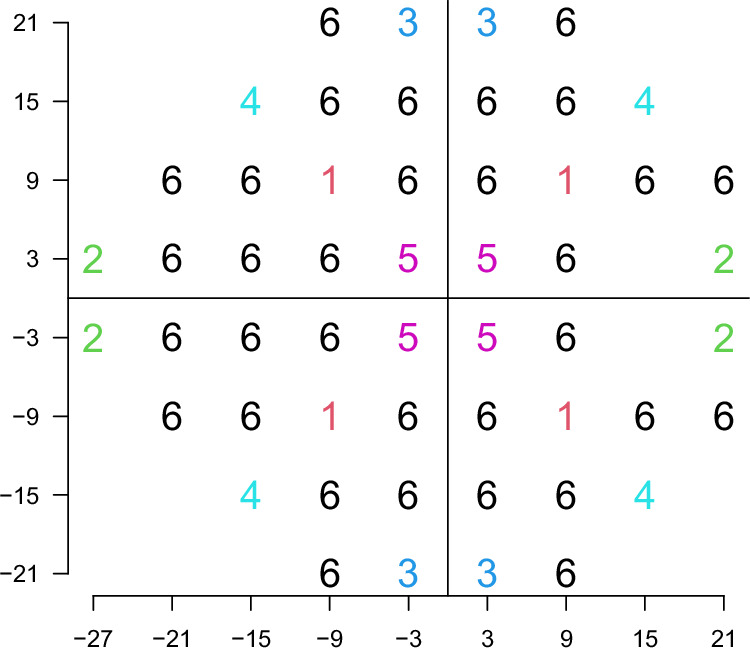
Location selection blocks for the right eye within the visual field. Testing begins at locations in block 1 (red), then progresses to block 2 (green), 3 (blue) and so on (see text for details). Within each block, locations are selected randomly for presentation. *X* and *Y* axes indicate degrees of visual angle.

The testing of quadrants and peripheral locations near meridians in the 24-2 pattern was prioritised by defining blocks of locations that should be tested in numerical order before moving to the next block. Locations within a block were randomly selected for presentation. Figure 2 shows the blocks used. At the beginning of the test, one presentation at each location in block 1 was given as a practice and then discarded from the analysis. Then, each location in block 1 received two presentations before being moved into block 6, effectively deferring any more presentations at these locations until block 5 was completed. Each location in blocks 2, 3, 4 and 5 received one presentation each before being moved to block 6. Once all locations had been moved into 6, incomplete locations were chosen in order of the largest distance between the remaining possible outcomes for the location until the test was terminated.

The particular locations shown in Fig. 2 are chosen to maximise detection and distinction of typical visual field loss archetypes [8, 9]. For example, if the test stops after eight presentations, two presentations have been made in each quadrant, allowing gross quadrantic and hemianopic defects to be detected. After 16 presentations, additional definition across the horizontal and vertical midlines is achieved. Each block is also represented in each quadrant to encourage spatial attention across the entire field so that testing is not focussed in one area, inviting a loss of fixation.

### Fast Test Logic

**Fig. 3:**
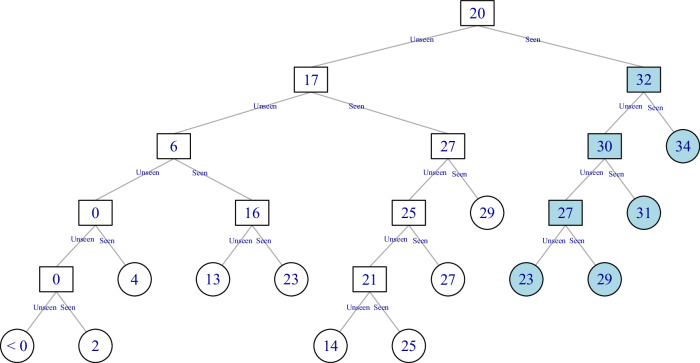
Decision tree as used for testing each location. Rectangular nodes indicate stimulus presentation values in decibels (dB), and circular nodes are the final measured threshold values in dB. The blue part of the tree is adjusted based on the eccentricity of a location (see text).

No spatial dependencies were created between locations in the test logic. Accordingly, each of the 52 test locations has its own procedure that it steps through, which can be described using a decision tree as shown in Fig. 3. Each rectangular node in the tree represents a stimulus presentation and, starting at the top, either the ‘Seen’ or ‘Unseen’ lines is followed when a response is received. The circles represent the final threshold value, if one is reached, with the current rectangle used as the threshold estimate if the test terminates before a circle is reached. As an aside, the lack of dependence between locations makes it easy to continue previous tests from a saved state.

As expected, most tests do not run to completion in children. Therefore, the test procedure was designed at each location to distinguish healthy from damaged locations early in the logic. The test was based on the binary decision tree generated using a standard Zippy Estimation by Sequential Testing procedure, with a bimodal prior similar to the one used in many previous studies (e.g., [10]). To shorten the procedure, the tree was ‘pruned’ repeatedly by removing any pair of leaves that were not separated by at least 5 dB. The final tree used at locations (±3, ±3) is shown in Fig. 3. Other locations use the same tree, except with a constant reduction to the blue nodes based on the rounded values of the hill of vision model from Fig. 12 of Pricking et al. [11]. If all 52 test locations in the visual field followed the shortest or longest path in the tree, then the range of presentations is between 156 and 312 for a single eye to complete testing.

### Presentation of Results

**Fig. 4:**
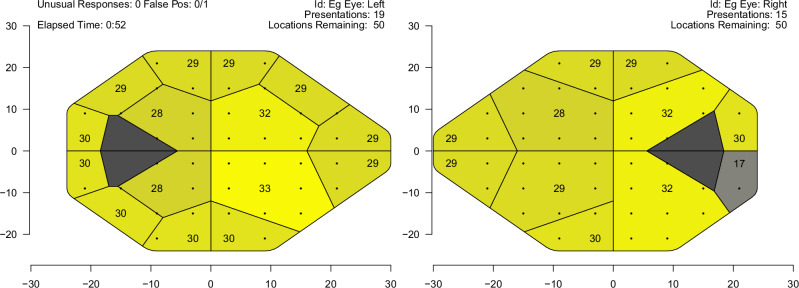
Example of the test screen shown to the perimetrist after 34 presentations (19 in the left eye and 15 in the right). Brighter yellow indicates higher sensitivity estimates. Numbers are only shown for locations that have had at least one presentation. Dots show the underlying 24-2 test pattern. The diagonal regions indicate the currently determined Voronoi tessellations [12] that divide the visual field into regions closest to each tested location, restricted by quadrant boundaries. The “locations remaining” indicates the number of 24-2 test pattern locations where the sensitivity estimate is not finalised.

After every presentation, an estimate was made of all locations in the field by taking the current value in the decision tree for locations that had at least one presentation, and the spatially closest value for locations that had not received a presentation. To emphasise which locations had received a presentation, the Voronoi Tessellation [12] was presented to the perimetrist, where untested locations are coloured the same as their nearest tested neighbour, restricted within visual field quadrants. An example is shown in Fig. 4; a screenshot of the Shiny interface (shiny.posit.co) built for the test.

### Engaging Embellishments

cSAP also included the addition of engaging graphics and sounds to motivate the children and keep their attention on the task. The test was designed as a series of ‘levels’. For each level, a different shape of fixation marker was used. The level consisted of 45 presentations in total, combined across the two eyes.

Every 10 presentations, the fixation marker changed colour. At the end of the level, a short audio tune played, and the fixation marker loomed to be very large and to have a smiley face. An illustration of the components is shown in Table 1. If the child was still engaged in the test after level 3 (total of 135 presentations), the levels continued (such that Level 4 was ‘circle’; Level 5 ‘square’ etc). Specific instructions provided to the children are detailed below.

**Table 1:**
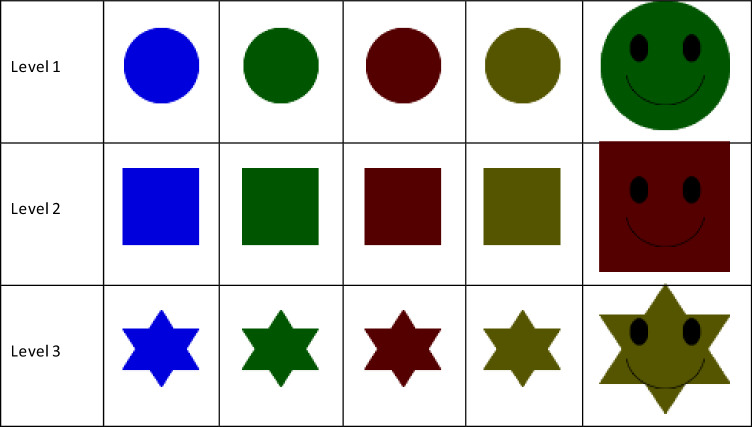
Fixation markers and end-of-level graphics for each level.

### Adult Group

Ten adults attending the Lions Eye Institute for their ophthalmic care were invited to participate in a single test session that occurred following their clinical care visit. Participants were recruited with a variety of visual field defect types and severity (see Table 2). Adults were tested using dichoptic presentation, resulting in both eyes being tested independently within the same test. The study was approved by the University of Western Australia Human Research Ethics Committee (UWA HREC 2024/ET000008), and participants provided written informed consent. Participants received a small reimbursement for their participation.

**Table 2:** Demographics of the adult eyes tested.

	Age (yr)	MD R (dB)	MD L (dB)	Eye disease
A01	30	0	−5.08	Retinal lesion
A02	62	−3.94	−2.27	Primary open-angle glaucoma
A03	75	−7.82	−6.36	Primary open-angle glaucoma
A04	34	−5.31	−10.45	Congenital myopia and secondary glaucoma
A05	66	−3.09	−12.78	Primary open-angle glaucoma
A06	61	+0.88	−10.14	Chronic angle closure glaucoma
A07	23	−31.70	−6.40	Congenital cataracts and glaucoma
A08	76	−1.85	−1.86	Chronic angle closure glaucoma
A09	80	−2.60	−5.87	Primary open-angle glaucoma
A10	71	−0.20	−1.60	Primary open-angle glaucoma

*dB* decibel, MD shows the Mean Deviation from the most recent Humphrey Field Analyser SITA test; MD R for right eye, MD L for left eye.

The adult cohort was used to validate that the cSAP algorithm does produce a somewhat useful visual field estimate if terminated early, by analysing the differences between the measured fields after each presentation compared with the final measured field for each of the 10 adults. The sum of the absolute differences (SAD) is reported between the 52 locations in the fields for cSAP as measured and for a simulated baseline procedure.

**Fig. 5:**
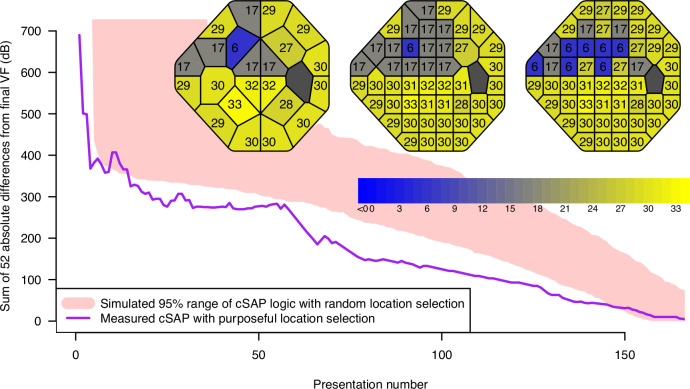
Results for testing adult eye A04. The sum of the absolute differences from the final visual field (VF) after each presentation is shown as the purple line; the pink shaded area indicates the 95% range over 1000 repeats of the same procedure, but randomly choosing locations to test. The inserted field plots show the measured field after 50, 100 and 150 presentations in the eye as labelled on the *x*-axis. The colour bar shows the shading used for each dB value in the fields. For this eye, the purple line is below the pink area in 138/167 presentations with a mean difference of 39.9. cSAP static automated perimetry in children, dB decibel.

The baseline procedure used the same decision tree logic with eccentricity corrections as cSAP for each location, but chose locations randomly for presentation rather than the purposeful placement strategy of cSAP. To compute a confidence range for the SAD for the baseline procedure, responses were simulated 1000 times for each eye using the baseline procedure, assuming that the final field of the participant is the true underlying threshold of the simulation. Response variability was modelled using the SimHenson simulation mode of the OPI [6], with default parameters and false response rates of 3%. In brief, this draws responses from a Cumulative Gaussian psychometric function whose mean is the assumed true threshold; standard deviation increases as threshold decreases and asymptotes at 0.03 and 0.97. Of the 1000 SAD values calculated at each presentation for the baseline procedure, the lower 95% were taken in order to report the number of presentations for which the SAD for cSAP fell below the lower 95% range of the baseline (see Fig. 5 for an example—the number of times the purple line falls below the red area was counted).

### Child Group

Children aged 4–9 years old (mean age: 6.9 years old) were recruited for this study (see Table 4). Recruitment and data collection were performed in ‘waves’ to permit revision of test procedures based on feedback from the child and/or parent. Two main testing waves occurred, aligning with Western Australian school holiday periods: June and September. Participants and their parents provided written consent and were phone-screened prior to attending for a single 30-min session at the Eye Health Centre of Western Australia. The study was approved by the University of Western Australia Human Research Ethics Committee (UWA HREC 2024/ET000008). Participants were also provided reimbursement for their participation. Participants were invited to return for a second session, following software improvements based on constructive feedback about the cSAP test. Parents and children mainly provided suggestions on how to engage the child during test time rather than the cSAP algorithm.

Seven children attended both sessions and four children attended for a single session (either in June or September). In the first session, participants underwent an ocular health screening by an optometrist prior to engaging with the prototype visual field to establish that the child had normal visual function as expected for their age. In the second session, participants tested the iterated version of the prototype visual field test, with the key differences being in the instructions given.

For the children, a game-like space-ranger narrative was used to explain the test: participants were asked to shoot down ‘asteroids’ using the Tempo button press by not directly looking at the asteroid (i.e., using their peripheral visual field). Perimetrists stressed the importance of paying close attention to the colour of their ‘planet’ (the fixation marker) which would change every 10 presentations throughout the test. Younger children were encouraged to shout out the colour change to provide feedback on attention. Fixation was also monitored throughout the visual field test via the in-built TEMPO camera, which enables the eye to be visualised. As such, throughout the test, perimetrists also encouraged the child to keep their gaze on the fixation target (‘planet’) rather than at the peripheral stimuli. Children were instructed to continue the visual field test to completion; however, they could stop when they were fatigued or disinterested.

To give some indication of accuracy, a ‘normal’ visual field was computed for the children by fitting a normal distribution to all threshold values >26 decibel (dB) at each of the eight eccentricities of testing (flipping left eyes to right for this purpose). This indicated a linear relationship of the mean normal dB values from 4.2*°* out to 27.2*°*, fitted with linear regression as normal dB values = 31.7−0.1 * (eccentricity in degrees). These were used to compute the Total Deviation values reported below.

### Ethics and Consent to Participate

Informed consent was obtained from all individual adult participants included in the study. All children had the study explained in an age-appropriate manner, including the use of a child-friendly information sheet. Children were given the opportunity to ask any questions and verbally assented to participate. Formal written consent after review of written study materials was provided by parents/guardians. All information and consenting materials provided to both adults, children, parents, guardians were approved by the University of Western Australia Human Research Ethics Committee.

## Results

### Validation on Adults

Figure 5 shows an example of the performance of cSAP (purple line) on one eye, compared with 1000 simulated repeats of the baseline procedure (pink area). In this eye, the estimate of the visual field at each presentation number after the 14th presentation has a lower SAD from the final field than the simulated procedure (purple line is below the pink area). For all eyes, cSAP is always below the upper 95% level of the baseline at all presentation numbers. This means that terminating cSAP at any number of presentations will return an estimate of the entire visual field that is never worse than the baseline procedure. Table 3 shows the number of presentations where the SAD of cSAP was lower than the 95% range of the baseline (purple lower than pink in Fig. 5) as a percentage of the total number of presentations for that eye. As can be seen, all the eyes had some point where termination would yield a more accurate estimate of the final field than the baseline, although eight of the 20 eyes had proportions <10%.

**Table 3:** Proportion of presentations where the sum of absolute differences (SAD) of Static Automated Perimetry in Children(cSAP) fell below the 95% range of the baseline comparator procedure.

Eye		Number of presentations where SAD for cSAP was below 95% CI baseline (count/total)		Mean difference in SAD across all presentations (dB)
Left	A01	2/ 134	2%	0.2
	A02	6/ 192	3%	2.3
	A03	127/ 168	76%	57.5
	A04	89/ 217	41%	21.1
	A05	106/ 193	55%	14.6
	A06	4/ 186	2%	0.8
	A07	4/ 185	2%	2.0
	A08	16/ 177	9%	0.5
	A09	73/ 154	47%	5.4
	A10	59/ 165	36%	3.5
Right	A01	23/ 108	21%	1.6
	A02	126/ 188	67%	24.5
	A03	177/ 206	86%	36.8
	A04	138/ 167	83%	39.9
	A05	10/ 178	6%	1.8
	A06	21/ 117	18%	1.3
	A07	114/ 223	51%	54.5
	A08	3/ 196	2%	0.6
	A09	6/ 152	4%	0.4
	A10	66/ 143	46%	10.8

A indicates an adult participant.

*CI* confidence interval, *dB* decibel.

**Fig. 6:**
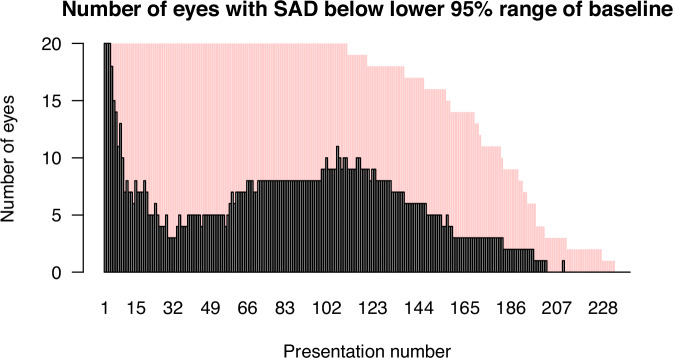
Number of eyes where the Static Automated Perimetry in Children (cSAP) procedure’s sum of the absolute differences (SAD) was lower than the 95% range of the baseline (black). The red bars indicate the total number of eyes remaining in the population at that presentation number.

Figure 6 shows for each presentation number, the proportion of eyes that had a lower SAD for cSAP than the 95% range of the baseline. When the test was terminated for any value less than 200 presentations, approximately 20–40% of eyes benefitted from the location placement logic of the cSAP algorithm.

### Testing on Children

The ages and numbers of presentations tolerated (before the child quit the task) and median Total Deviation are shown in Table 4. At their first visit, all but one of the children (4 years of age) tolerated the test to some degree, and all children aged above 5 years returned some measure of the visual field that would be reasonable to assume as undamaged (Median Total Deviation above −1 dB). The two youngest children performed much better at their second visit, tolerating an extra ‘level’ of testing and returning normal fields. The spatial depiction and dB for all final fields are provided in Online Resource A.

**Table 4:** Age, number of visual field presentations and median Total Deviation at test completion or termination for the children (sorted by age).

				Median total deviation (dB)			
		Number of presentations		Left eye		Right eye	
Child (K)	Age (yr)	June	September	June	September	June	September
K02	4	35	74	**−12.56**	−0.23	**−11.88**	−0.18
K07	5	36	78	**−12.56**	−0.46	**−11.88**	−0.37
K05	6	108	105	−0.37	−0.98	−0.37	−0.46
K11	6	–	139	–	−0.37	–	−0.23
K01	7	218	78	1.06	0.11	1.44	0.11
K04	7	136	159	0.02	0.06	0.06	0.02
K08	7	–	187	–	0.57	–	−0.3
K09	7	–	187	–	0.7	–	−1.11
K06	8	115	–	−0.37	−	−0.3	–
K03	9	112	*217	−0.11	1.77	0.02	1.89
K10	9	–	107	–	−0.23	–	0.02

The test labelled with an asterisk was fully completed. Bold values are clearly outside normal limits. *dB* decibel.

Of the six children who performed the test twice, four had significantly more presentations, one less presentations and one about the same number. It is difficult to ascertain whether this was due to greater familiarity with the testing protocol, perimetrists, the environment, whether the perimetrists were better at delivering instructions or whether the mild alterations to the graphics based on feedback received in the first session captured the child’s attention better. For younger children, such as the 4-year-old involved in this study, having a smaller inter-pupillary distance precluded them from utilising the dichoptic version of the test. Thus, K02 was retested monocularly with an eye patch (K02).

At the end of the test session, both the children and their parents/guardians were asked for feedback regarding the test procedure. Specifically, the children were asked what they liked or did not like about the test; parents were asked what they thought about their child’s engagement with the task and whether they had any recommendations on the test. Constructive comments from children and parents were mainly about improving instruction, engagement and ergonomics of the visual field test and testing conditions. As a consequence of this feedback, for the testing in September 2024, children were instructed using a visualisation presentation in Microsoft PowerPoint (Microsoft.com).

## Discussion

This paper describes a method for testing visual fields in children that was designed to enable SAP to be completed, or to at least to achieve some estimate of the visual field if the test is terminated early. In general, the children engaged well with the task, with the exception of the very youngest child (aged 4 years) who struggled with maintaining task attention. This test procedure may also have advantages for testing adults with cognitive challenges or others who may need to abort a test prior to completion.

Conventional visual field tests used in children include confrontation or kinetic perimetry with a traditional Goldmann perimeter [2]. Confrontation testing is often used in the screening of visual field loss with relatively high specificity but has well known limitations in sensitivity. If a child disengaged from the current perimetry task after the initial quadrant testing, a similar outcome would be expected. Goldmann kinetic perimetry is a reliable option for paediatric perimetry over 5 years of age [13] with normative data established in children [14]. However, the instrument is no longer commercially available. While other kinetic perimetry testing, such as with the Octopus device (haag-streit.com), is feasible [15], it relies heavily on an experienced examiner to perform and document the assessment, which may limit testing to specialty clinics.

In addition to investigating how children perform on commercial perimeters, numerous research groups have tried other non-traditional methods for evaluating peripheral visual sensitivity. An example is ‘Saccadic Vector Optokinetic Perimetry’, which is a suprathreshold method of SAP that measures the response of the child via eye-tracking rather than requiring them to press a button [16, 17]. In short, if a saccade is performed in the direction of the stimulus, then it is assumed to be have been seen. Other techniques include the development of computer games [18], including some with complex storylines [19]. Both approaches involved suprathreshold visual field assessment, although frequency of seeing curves were also attempted in one study with some success [19].

The present study concentrated on SAP as the method for visual field assessment because, if reliable results can be achieved, it enables the child’s visual sensitivity to be followed longitudinally into adulthood using similar, standard clinical methodologies. This may be particularly relevant for the treatment and monitoring of childhood glaucoma [20]. Currently, there is no consensus on the optimal strategies for visual field assessment in children, and significant variance exists regarding the use and frequency of perimetry [21, 22]. A child-friendly version of SAP that enables direct transition into an ‘adult’ test as the child ages may increase the use and utility of visual field assessment in children. The design of the current approach has this in mind, where the decision tree in Fig. 3 can be extended (unwind some pruning) if further precision is required in adults.

This approach to location selection in cSAP has some similarities to previous methods; in particular, there are several investigations that use large databases of previously measured visual fields and machine learning to choose the location presentation order [23–25]. Unlike these previous designs, the present study did not rely solely on a data-driven approach to selecting locations at which to present but also injected clinical experience and other design factors, such as balancing spatial attention. In the visualisation of intermediate results to the perimetrist, Voronoi tessellation restricted by quadrants was chosen (Fig. 4), but note that disease-specific variants of custom visualisations for intermediate results might be useful in certain clinical situations. For example, the tessellations could be permitted to cross the vertical midline if the focus of the testing is glaucoma, and could possibly be influenced by known retinal nerve bundle trajectories.

In this manuscript, a normative database was approximated with a linear fit to the mean of threshold values greater than 26 dB, split by eccentricity in degrees. However, with more data, it may make sense to split normative values by both decision tree (hill of vision correction) and the number of presentations used at a location. The reasoning behind this suggestion is evident from the decision tree in Fig. 3. If one presentation is used, then the normative value can only be 32 dB using this tree. If two presentations are used, then the normative average will be a weighted sum of 27, 30 and 34 dB; for three presentations, a weighted sum of 29, 27 and 31 dB; and so on. It may be that the differences between these values are small and a single summary normative value may suffice, but it is noted here for future work.

In conclusion, this project aimed to design and conduct proof-of-concept evaluation of a child-friendly version of SAP. The child-friendly design considered hardware for child-friendly ergonomics, thresholding algorithm design and some gamification to encourage participation and engagement. The data from adults and associated computer simulation assessment demonstrate that terminating early with this algorithm will typically return a more accurate estimate of visual field status than a reference comparison algorithm. Children were engaged with this prototype version of the test. Further evaluation in larger groups of children with a variety of vision disorders is a necessary next step, along with the collection of normative data in children.

## Supplementary information

supplementary information

## Data Availability

Individual data for the perimetry tests are provided as online supplementary material. All other data are available from the authors upon reasonable request.
