# Supplementary material for: Devising and Evaluating an Adaptive Static-Automated Perimetry Test for Children: A Feasibility Study
Source: Ophthalmic Physiol Opt. 2026 Mar 16;46(2):322–30. doi: 10.1007/s44402-026-00049-9 (PMC13369772; doi:10.1007/s44402-026-00049-9)
Supplement: Supplementary file 1 — supplementary information [file 44402_2026_49_MOESM1_ESM.pdf]

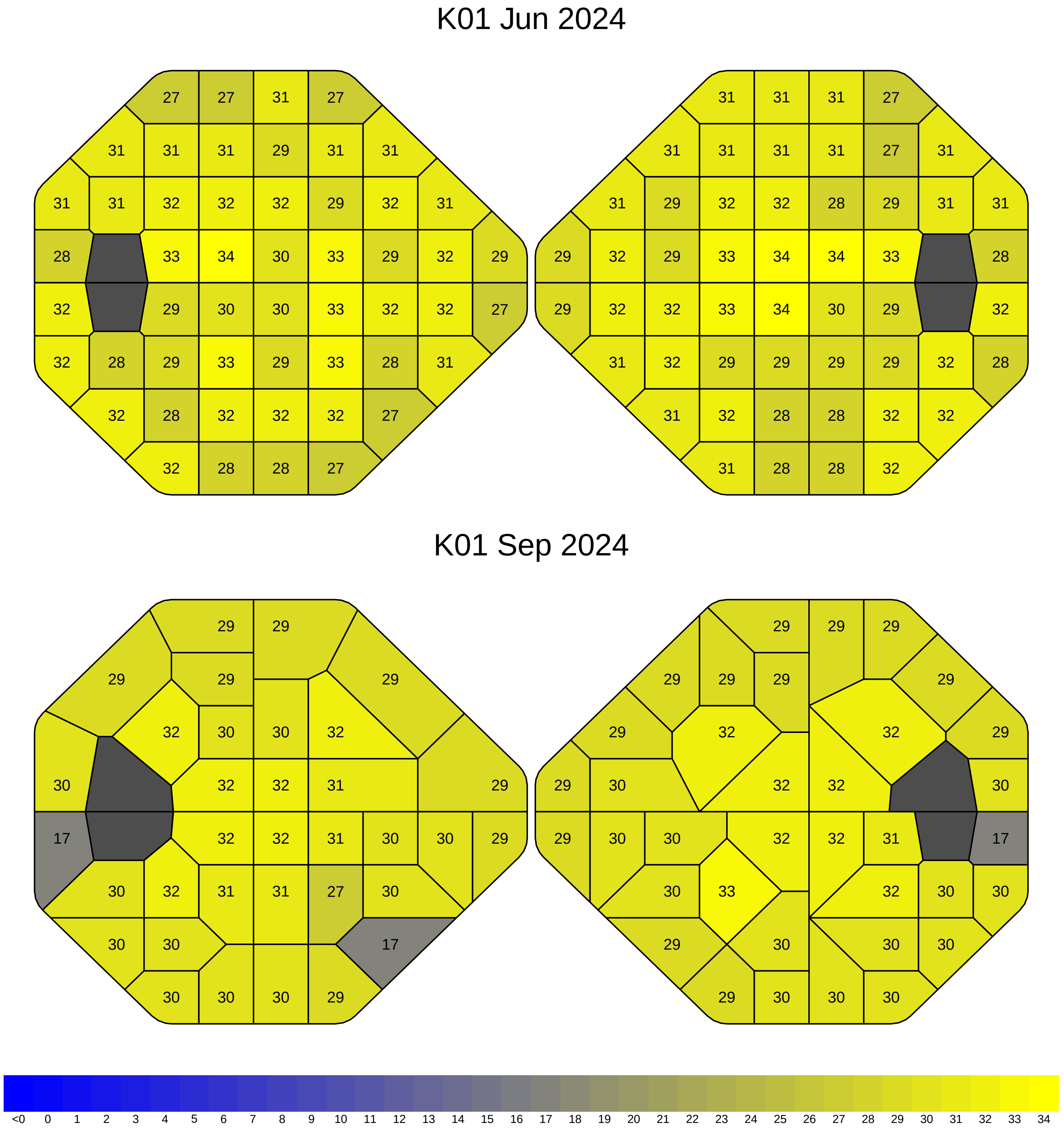

All figures are presented using Voronoi tesselation separately in each quadrant.

K02 Jun 2024

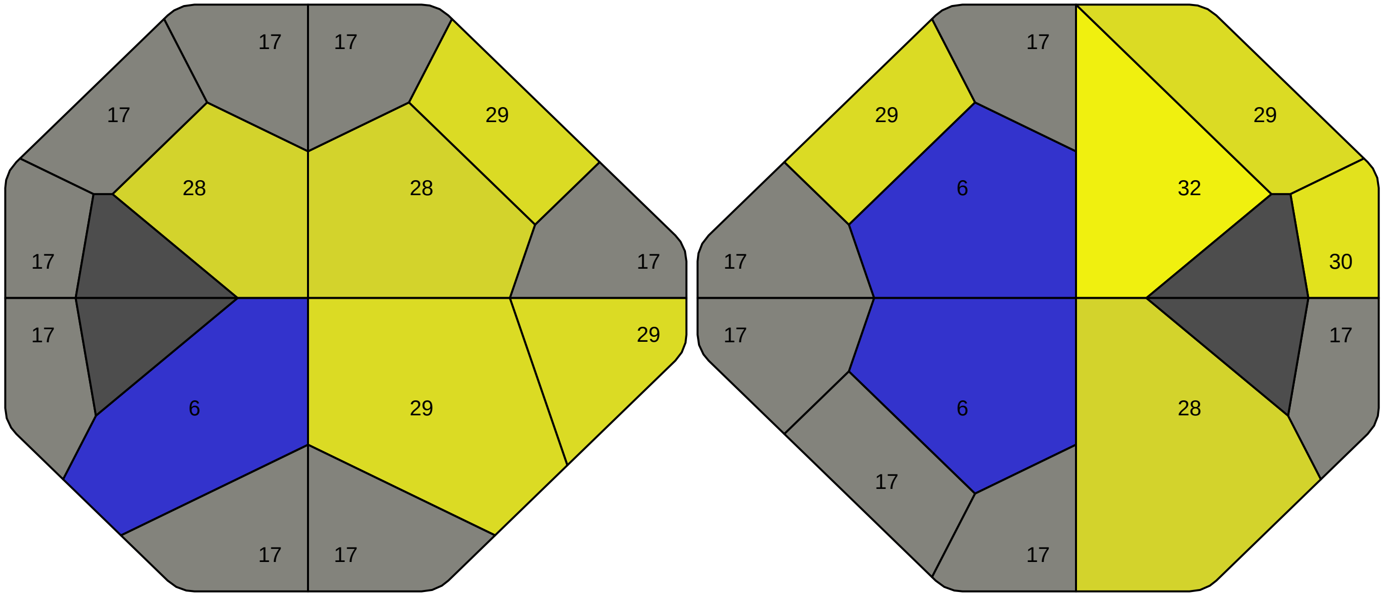

K02 Sep 2024

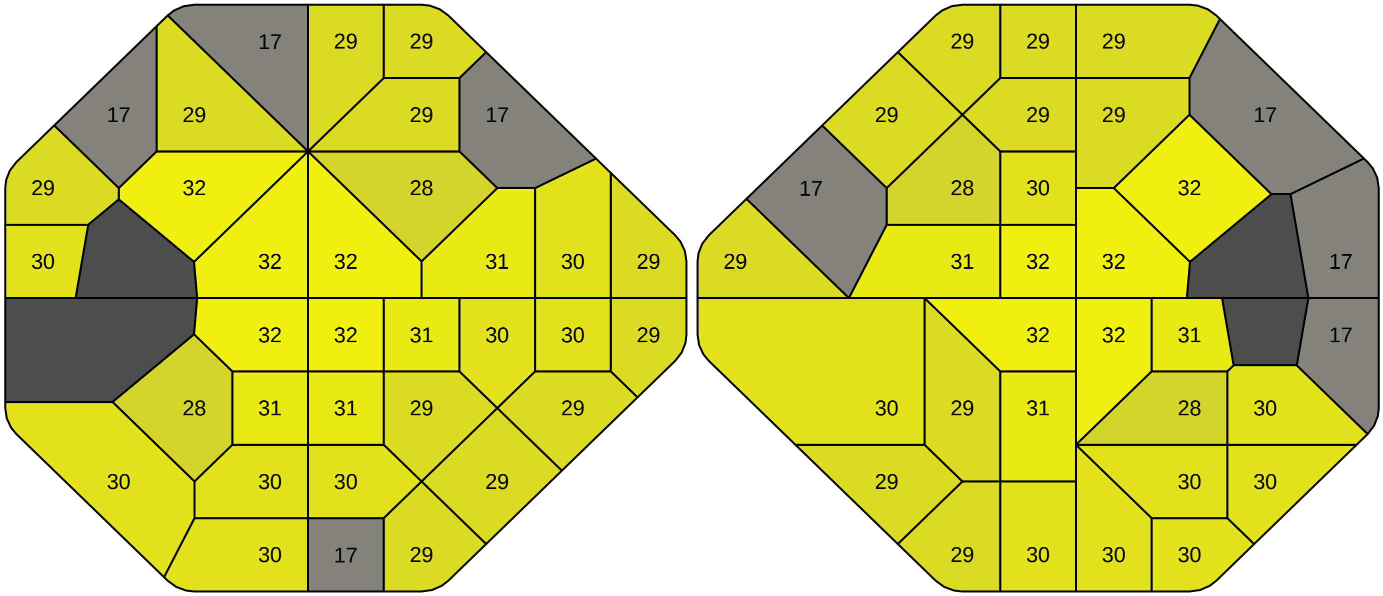

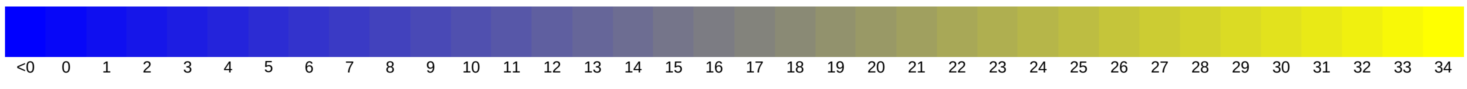

Note the first response to locations (+3, +−21) in the right eye (Jun), location (−21, −3) left eye
and (−27, −3) right eye (Sep) was faster than 150 ms and so was re−queued for a repeat presentation by the test logic.
Due to a software bug, the repeat presentations were not necessarily re−presented in their correct block.

K03 Jul 2024

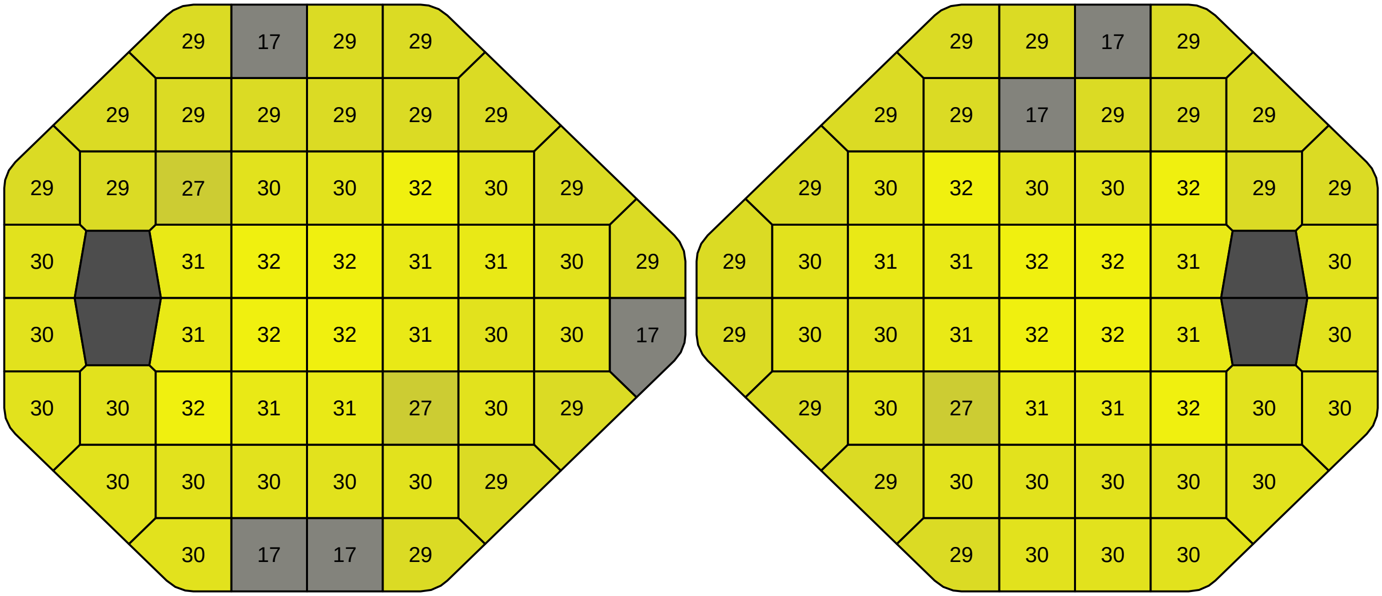

K03 Sep 2024

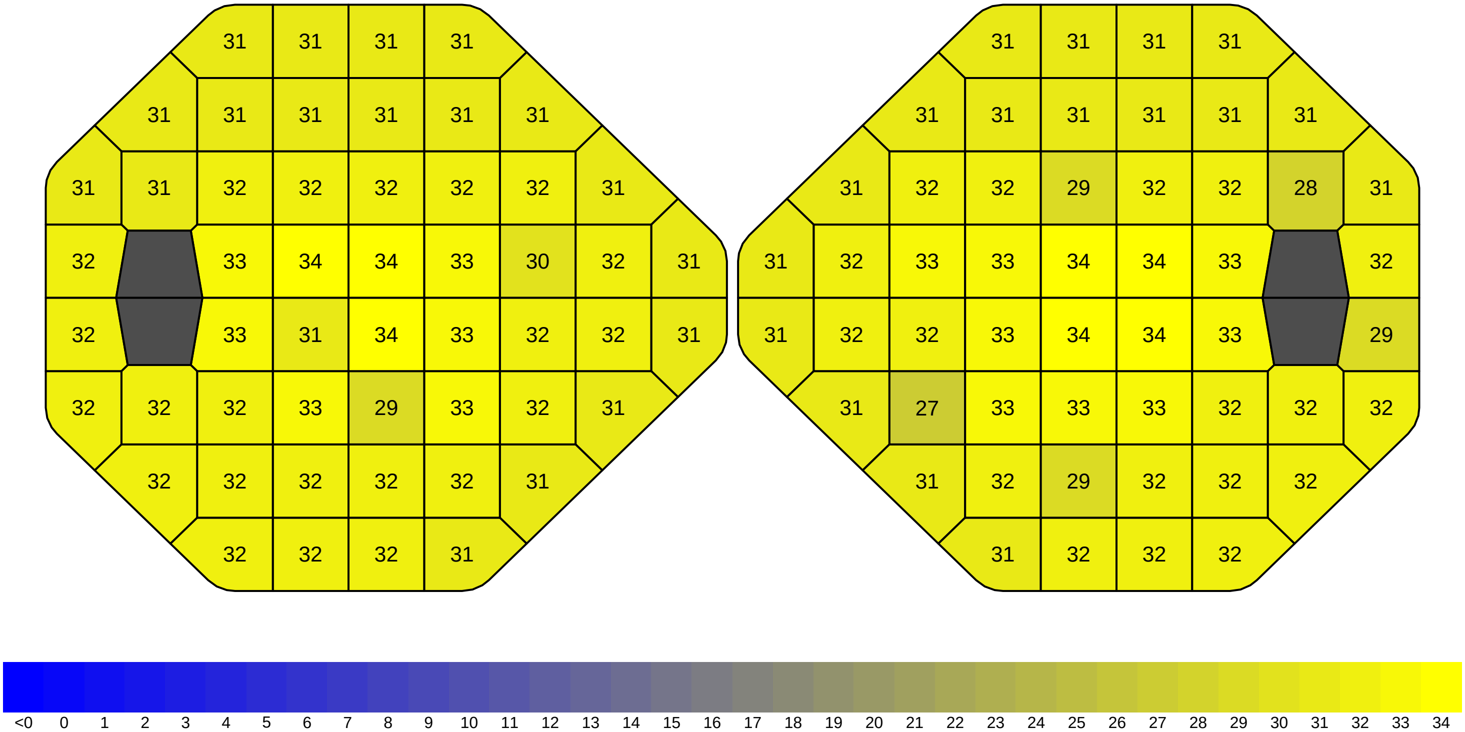

K04 Jul 2024

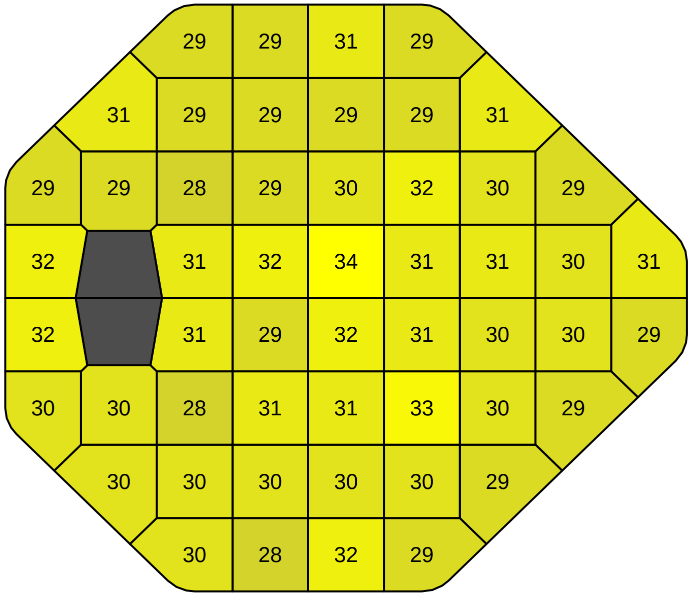

K04 Sep 2024

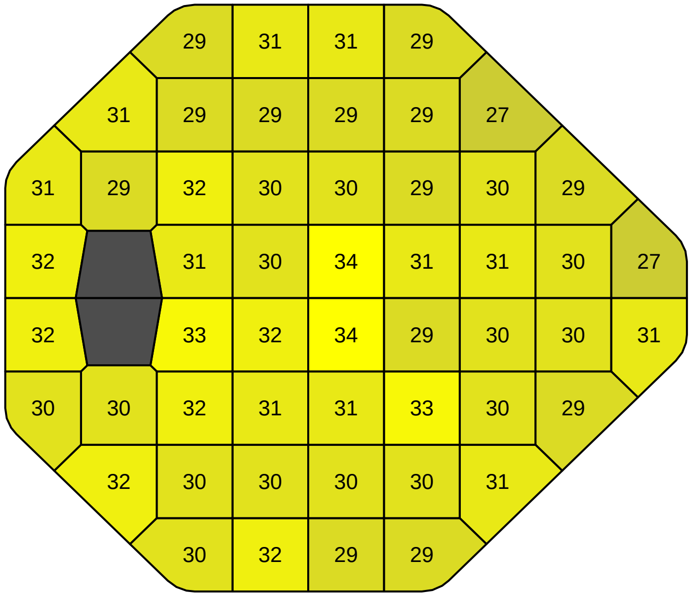

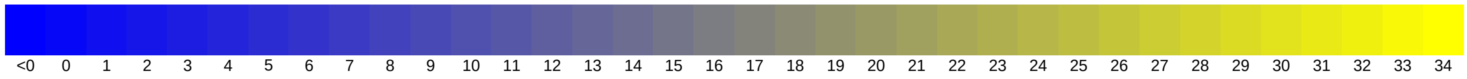

K05 Jul 2024

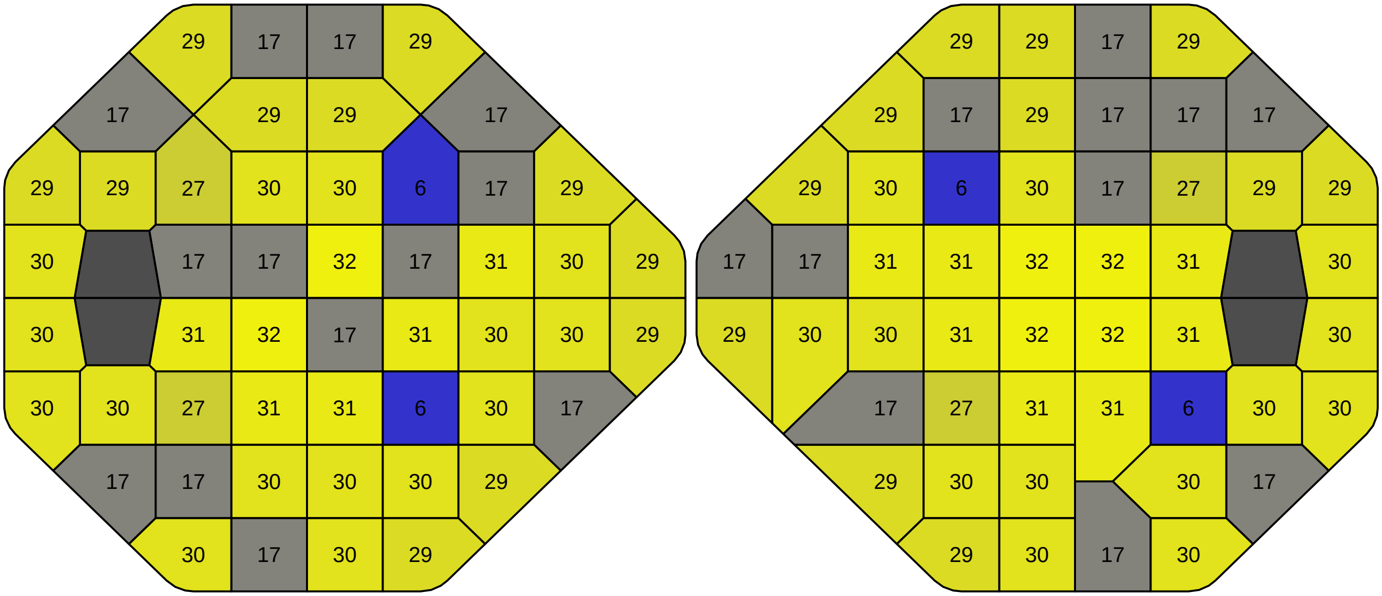

K05 Oct 2024

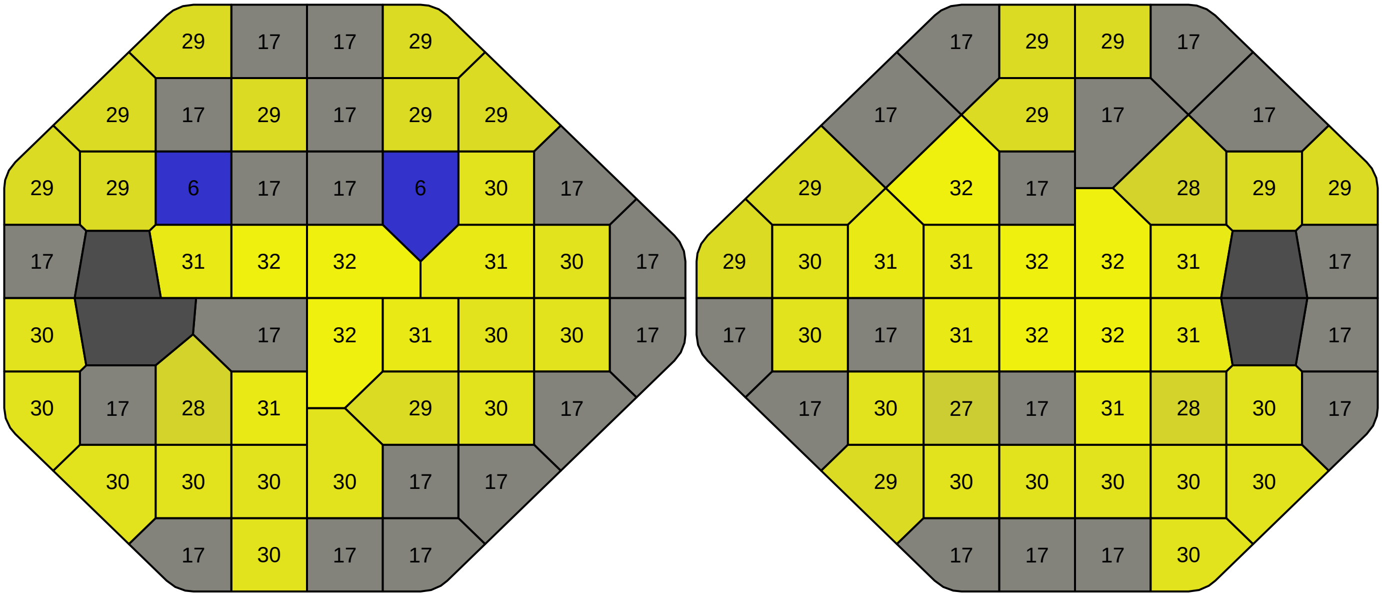

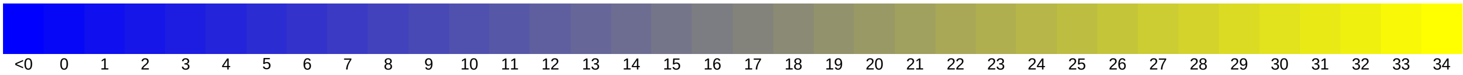

K06 Jul 2024

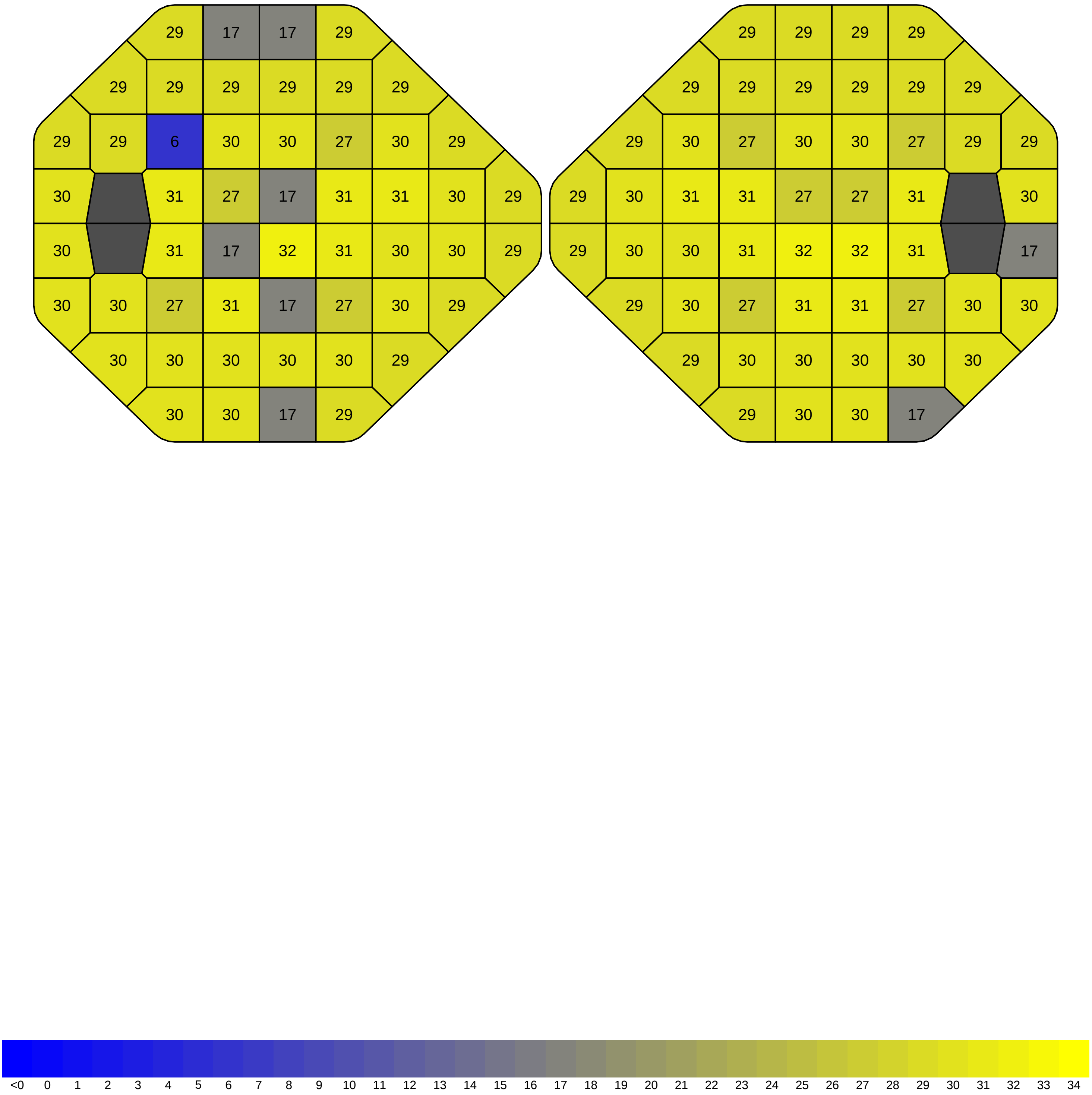

29 17 17 29
29 29 29 29 29 29
29 29 6 30 30 27 30 29
30 31 27 17 31 31 30 29
30 31 17 32 31 30 30 29
30 30 27 31 17 27 30 29
30 30 30 30 30 29
30 30 17 29
29 29 29 29
29 29 29 29 29 29
29 30 27 30 30 27 29 29
29 30 31 31 27 27 31 30
29 30 30 31 32 32 31 17
29 30 27 31 31 27 30 30
29 30 30 30 30 30
29 30 30 17
<0 0 1 2 3 4 5 6 7 8 9 10 11 12 13 14 15 16 17 18 19 20 21 22 23 24 25 26 27 28 29 30 31 32 33 34

K07 Jul 2024

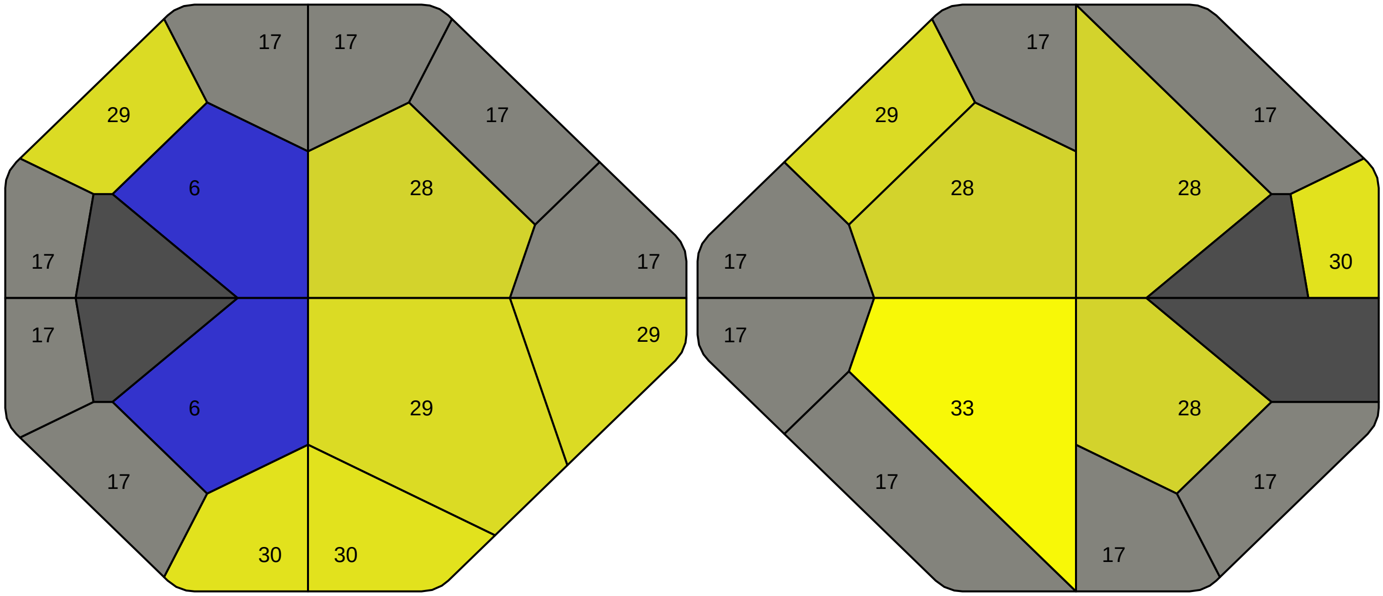

K07 Sep 2024

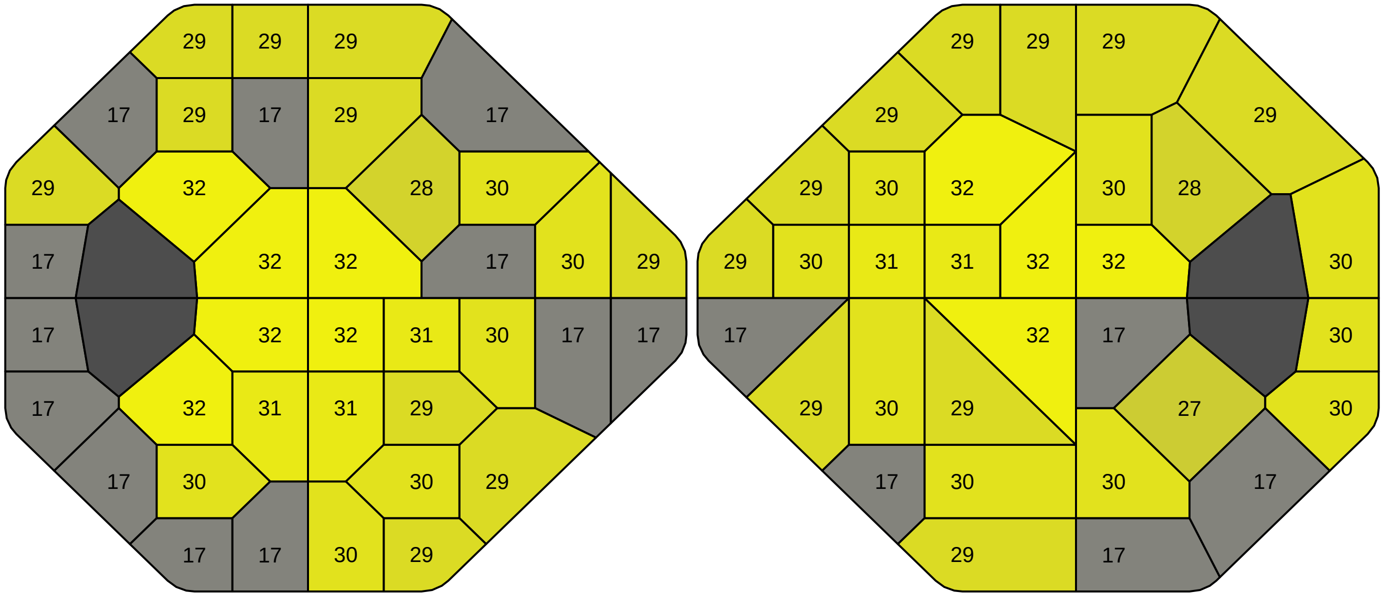

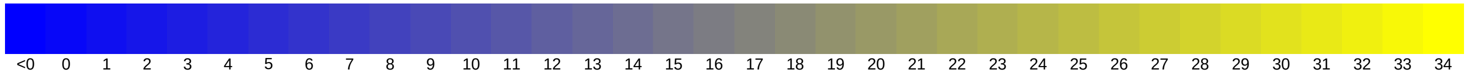

Similar to K02, the first response to locations (+3, +21), (−3, −21) and (21, −3) in the right eye (Jul) was faster than 150 ms and so was re−queued for a repeat presentation by the test logic but then due to the bug, the repeat presentations were delayed.

K08 Oct 2024

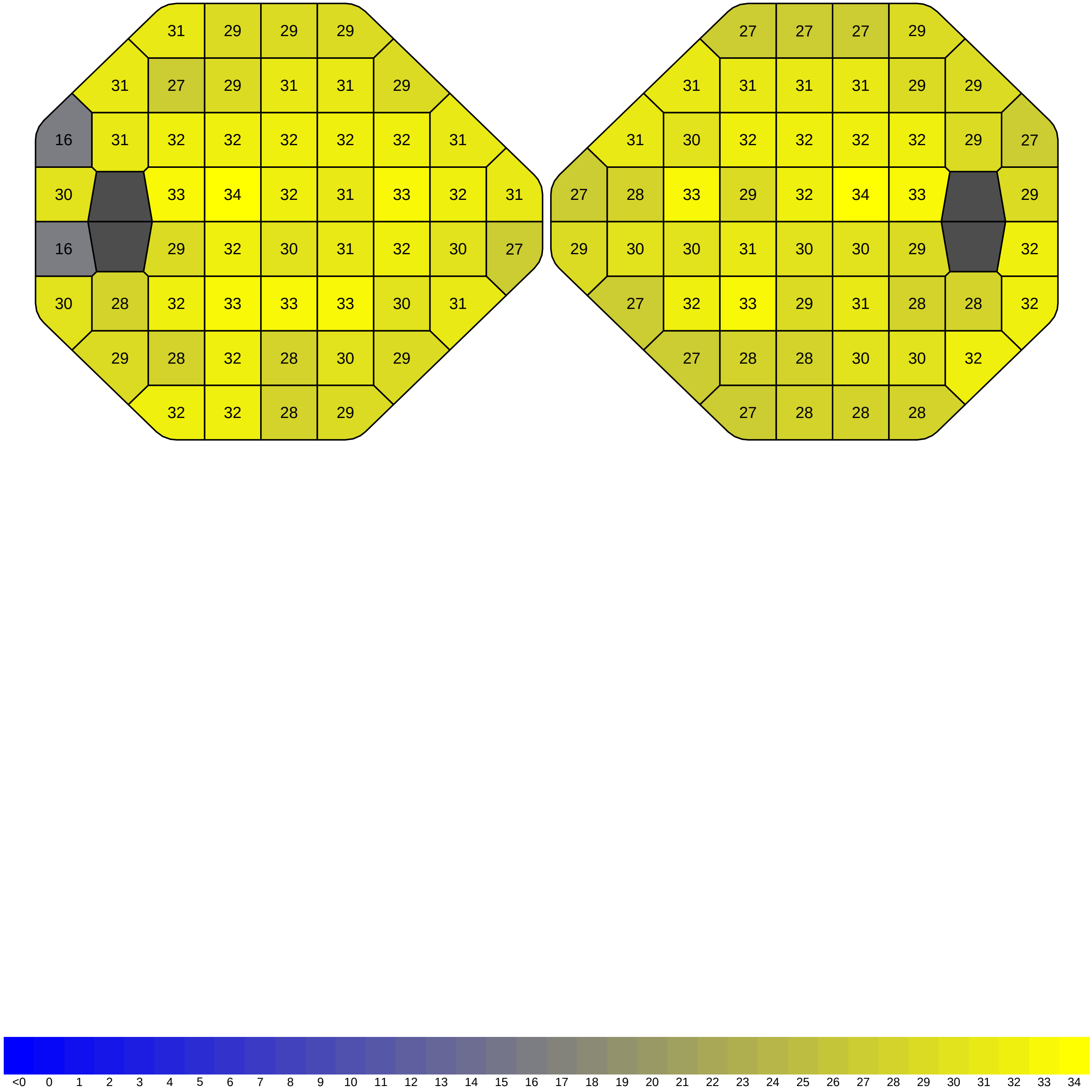
31 29 29 29
31 27 29 31 31 29
16 31 32 32 32 32 32 31
30 33 34 32 31 33 32 31
16 29 32 30 31 32 30 27
30 28 32 33 33 33 30 31
29 28 32 28 30 29
32 32 28 29
27 27 27 29
31 31 31 31 29 29
31 30 32 32 32 32 29 27
27 28 33 29 32 34 33 29
29 30 30 31 30 30 29 32
27 32 33 29 31 28 28 32
27 28 28 30 30 32
27 28 28 28
<0 0 1 2 3 4 5 6 7 8 9 10 11 12 13 14 15 16 17 18 19 20 21 22 23 24 25 26 27 28 29 30 31 32 33 34

K09 Oct 2024

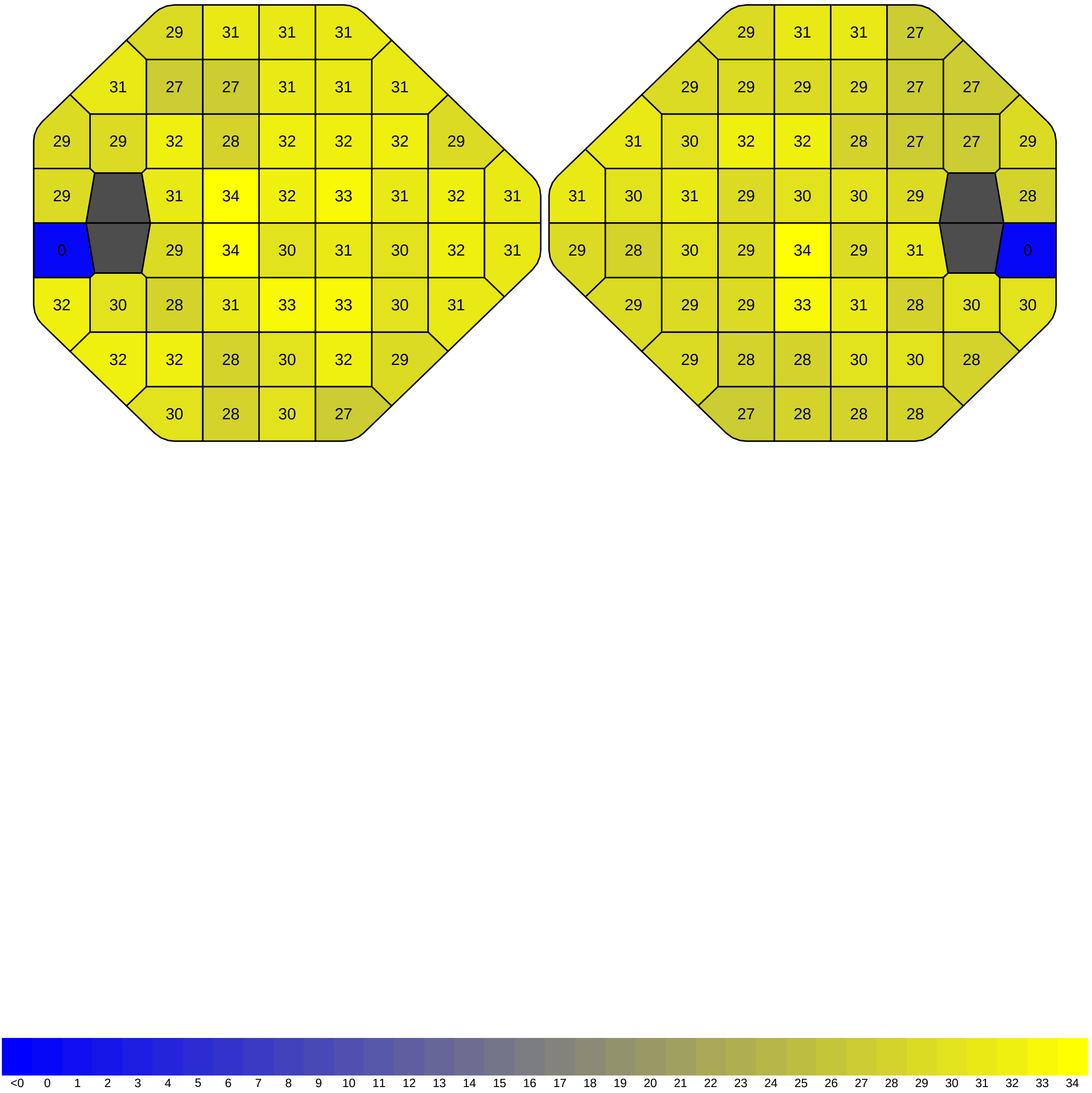

29 31 31 31
31 27 27 31 31 31
29 29 32 28 32 32 32 29
29 31 34 32 33 31 32 31
0 29 34 30 31 30 32 31
32 30 28 31 33 33 30 31
32 32 28 30 32 29
30 28 30 27
29 31 31 27
29 29 29 29 27 27
31 30 32 32 28 27 27 29
31 30 31 29 30 30 29 28
29 28 30 29 34 29 31 0
29 29 29 33 31 28 30 30
29 28 28 30 30 28
27 28 28 28
<0 0 1 2 3 4 5 6 7 8 9 10 11 12 13 14 15 16 17 18 19 20 21 22 23 24 25 26 27 28 29 30 31 32 33 34

K10 Nov 2024

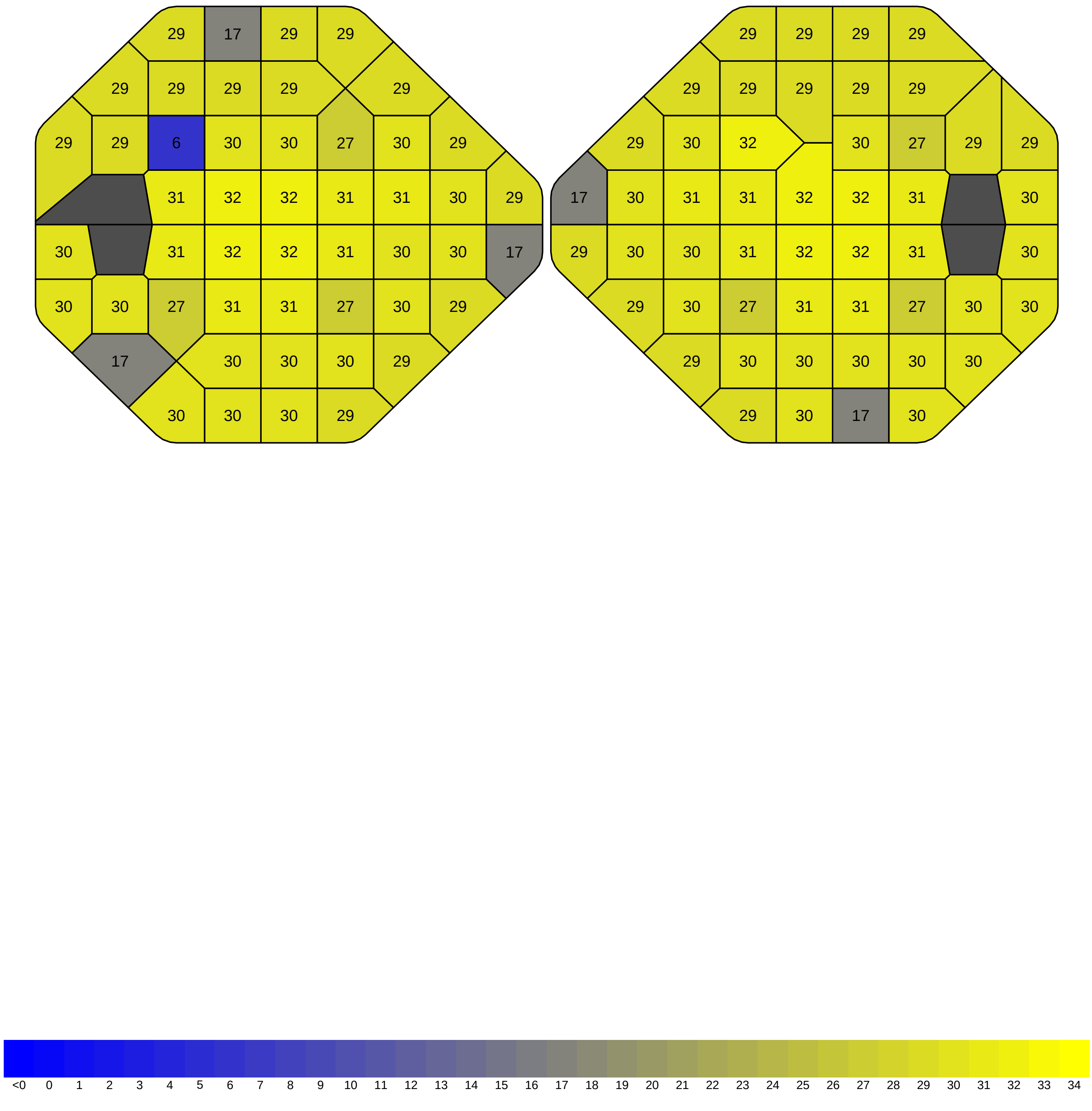

Similar to K02 and K07, the first response to location (−21, +3) in the left eye was faster than 150 ms and so was re−queued for a repeat presentation by the test logic but then due to the bug, the repeat presentation was delayed.

K11 Nov 2024

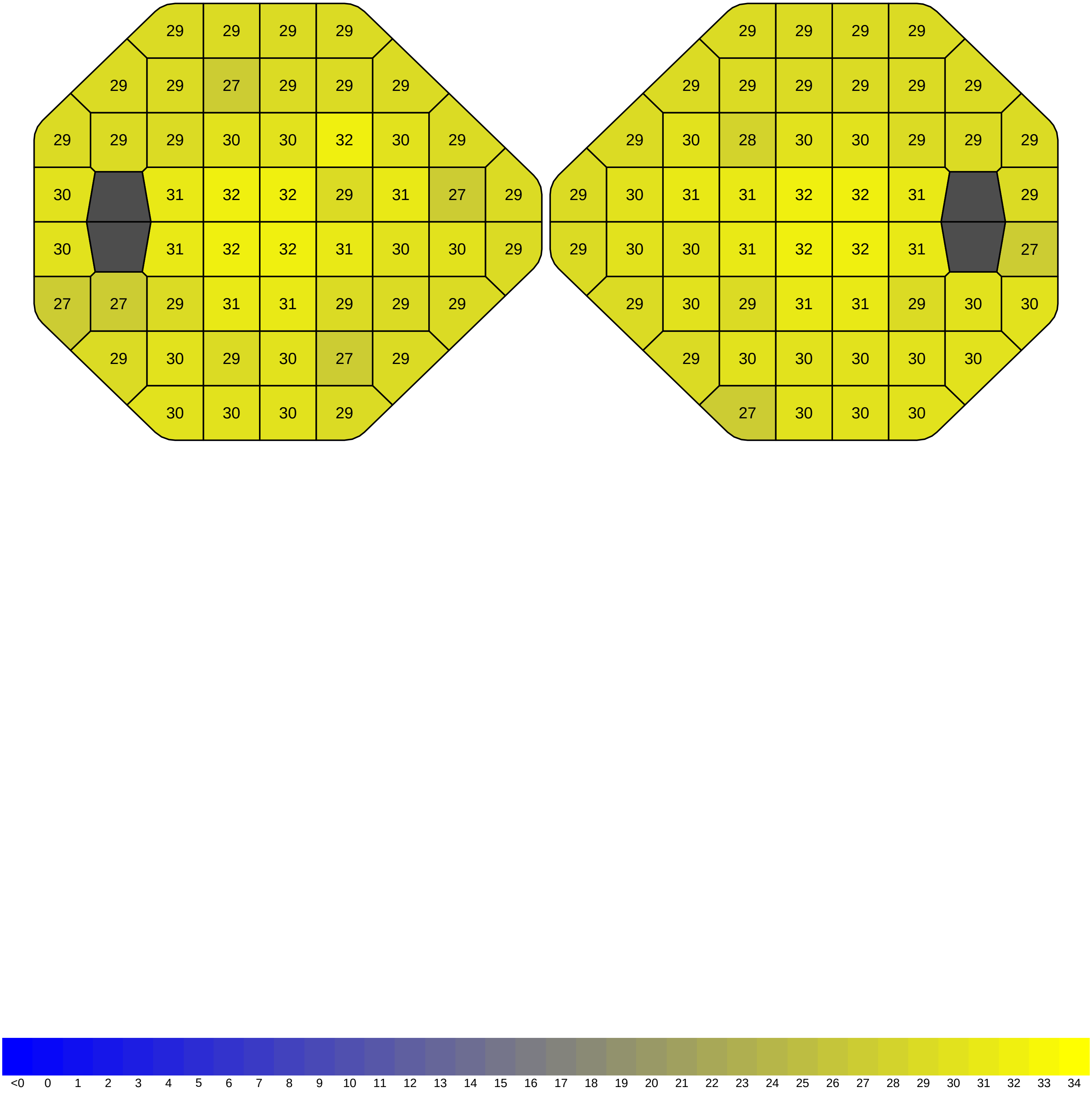

29 29 29 29
29 29 27 29 29 29
29 29 29 30 30 32 30 29
30 31 32 32 29 31 27 29
30 31 32 32 31 30 30 29
27 27 29 31 31 29 29 29
29 30 29 30 27 29
30 30 30 29
29 29 29 29
29 29 29 29 29 29
29 30 28 30 30 29 29 29
29 30 31 31 32 32 31 29
29 30 30 31 32 32 31 27
29 30 29 31 31 29 30 30
29 30 30 30 30 30
27 30 30 30
<0 0 1 2 3 4 5 6 7 8 9 10 11 12 13 14 15 16 17 18 19 20 21 22 23 24 25 26 27 28 29 30 31 32 33 34
